# Extensive Occlusal Restorations

**Published:** 1914-09-15

**Authors:** W. D. N. Moore

**Affiliations:** Chicago


### EXTENSIVE OCCLUSAL RESTORATIONS.

BY W. D. N. MOORE, L.D.S., D.D.S., CHICAGO.

NOTE.—This is an extract from a paper presented to the Canadian Dental Association and printed in the DOMINION DENTAL JOURNAL.

The usefulness and comfort of many mouths of older patients have been almost, and often entirely crippled as a result of abrasion of the teeth. It may be very marked in mouths where no loss of teeth has been suffered, but it is usually accompanied by the absence of a few or many teeth. In any event the restoration of such a mouth to its full usefulness has been no small task, if success is to be attained. The very fact that tooth enamel and dentine have been worn away, either partially or to the full extent of the natural crown, is evidence that a mighty force is to be brought to play upon any restoration that is to be made. To witness the abrasion of the entire dentition is an impressive lesson of what strength must be put into our work if we are even to attempt competing with nature whose substance was not equal to the strain. If we are to undertake to give such a patient comfort and service, it ill affords us to be faint-hearted. I know of no cases demanding more skill for the operator than these. Such cases are too rarely attempted, due presumably to the great extent of work involved, together with a doubt in the operator's mind as to the outcome. If would be a serious misfortune to patient and dentist if failure resulted, but inspiring and strengthening to the operator beyond anything that he can experience if success is accomplished; and it can be.

Before the days of casting, unless crowning of every tooth in the mouth was the procedure, little could be hoped for in a permanent way. Casting has offered a successful solution of the problem and when properly carried out in all the details involved renders possible a great service to mankind.

When teeth are not missing and the abrasion has involved the total loss of the natural crown of the tooth, inlaying with a metal of much resistance should be employed. The importance of a hard metal cannot be overlooked and its use is imperative. Pure gold or even 22 k. gold would invite failure. The question of the proper metal to be used for this purpose must be carefully considered. It is well known that pure gold can be alloyed with many metals to offer a surface much harder than that offered by pure gold or 22 k. gold. Silver and copper are commonly used for the purpose of alloying a pure gold. But the trouble arises that gold alloyed in this manner will not cast as accurately as pure gold, and an ill fitting inlay results. Of the two evils which is to be chosen? The answer is, neither one; since it is possible to have an inlay that will fit the cavity perfectly and at the same time present an occlusal surface that is sufficiently hard to withstand the severest strain from opposing teeth.

To accomplish this, adapt the wax to the cavity proper and including all the margins thereof, but do not attempt a full restoration of occlusal surfaces and not necessarily of the interproximal surfaces. This is then cast in pure gold in order to get perfect adaptability of metal to tooth structure. This casting is then placed in the cavity, burnished and polished to all the margins of the cavity. When this has been done remove it, and after drying the gold thoroughly warm it sufficiently so that inlay wax will adhere. Add sufficient wax to make contour and the restoration desired on the occlusal part of the tooth. This is determined by the extent of abrasion. In any event, sufficient wax should be added so as to restore a crown of proper length for the individual seeking service, and can only be arrived at from a knowledge of tooth anatomy together with the indication necessary to establish a new occlusion. This mass of wax now adhering to the gold already fitted to cavity should be carved in detail to resemble the natural crown. The whole mass can now be invested and second cast made. The metal with which this second casting is to be made is of vital importance. After making many experiments, it is the writer's belief that no metal added to pure gold to give a hard surface and yet cast next in accuracy to pure gold, surpasses platinum for this purpose. It can be used in a varied percentage, depending upon the case. In cases such as we are now considering, ten to fifteen per-cent. would give good results. Clasp metal is very good to be used for this purpose, but, peculiar as it may seem, after the first or second casting the metal refuses to cast at all well. It may be claimed that the second casting will unite imperfectly with the first, due to a deposit left on the gold. Any doubt in this respect can be relieved by flowing a very small amount of solder on the proximal surfaces at the point of contact of the two metals. When polished thoroughly, an inlay fulfilling all the requirements demanded is obtained.

Commencing with the first molar, the bicuspids and other molars on the same side in the same arch should have inlays made in a similar manner before any cementing is done. Precaution must be taken to establish the proper occlusal plane when these inlays are in place. Also carving in a detailed manner should be given to each inlay, the proximal surfaces of which should extend well to gum line, even to within the free margin of it.

When all the inlays for the four or five posterior teeth have been made and set in place, but not cemented, wax these in their correct relation to each other while each is properly seated in its cavity, and remove and invest. Having been careful to gain the correct contact of all these inlays, they can be attached to each other with the smallest amount of solder. When cleaned and polished, these should be cemented in place and the four or five opposing teeth treated in a similar manner, being careful to open the bite about the right distance and establish correct anatomical occlusion with teeth already inlayed. I have mentioned the observance of care in carving accurately to tooth form, and I repeat because of its importance. Some may strenuously differ with the idea of assembling these; but it has been your essayist's experience, especially in cases where extreme pressure was exerted, that the planes made by our carving, no matter how carefully done, will frequently cause in the course of time a moving of the teeth that disturbs their contacts. It is true that carvings can be made so accurately as would apparently overcome this shifting, however small, but no operator can ascertain all the movements of the mandible, which is an important factor in this respect. Besides, experience in attaching these inlays and even crowns where used instead of inlays, gave such favorable results as to highly commend its practice. The mighty force occurring in many mouths has been sufficient to move the natural crowns apart, and before old age was nearly reached wide spaces existed. The disturbance to the individual mobility of these teeth has never made any effect on the minds of patients, according to their opinion in questioning them concerning the point. In fact, the expression of strength and firmness has been gained from remarks regarding assembling the work. "In union there is strength" and it seems to be as true here as in other things. To complete these extensive cases, usually the six anterior teeth of the upper mouth require some attention, but seldom the lower. They may or may not be as much worn down as the posterior teeth, and when abrasion is not evident on the incisal edges, deeply worn grooves are to be found along the lingual surfaces.

Inlaying or crowning depends upon the condition prevailing. Crowning very frequently is indicated, especially with the four incisors, and inlays on the tips of the cuspids. It is in such cases as these that the operative and prosthetic branches of dentistry must be closely allied to bring about the greatest possibilities. Where abrasion of the teeth has been most extensive, usually several of the teeth are found missing in one or both arches, subjecting few teeth to the wearing pressure of mastication. Where this condition is to be found artificial substitutes must be supplied to establish an unbroken occlusal plane. Crowns or bridges may have to be used instead of inlays, but the same principles must be employed in the most careful detail. It requires inexhaustible patience and perseverance, but the possibilities offer a most gratifying reward in the achievement that may be accomplished. Patients are most grateful, and have plainly stated on more than one occasion that they were not aware such results were possible in dentistry. This is not only good to those concerned in the undertaking, but it has the effect of placing a higher respect for the skill of our profession, which is most helpful to all engaged in it.

When these extensive cases first present themselves for treatment, little, if any suggestion of procedure should be given to the anxious patient—and anxious they usually are. In fact. the careful operator cannot tell, himself. This can only be gained from good plaster models, which should invariably be made of the mouth before any attempt is made to restore it to usefulness. Besides, an accurate chart should be made showing the cavities in the teeth, if any, and all fillings or other dental work that may have been already inserted. With good models and a chart, a careful study can then be made and the work outlined, and if undertaken adhered to very closely. As has been stated previously; it is here that the treatment of one or two teeth must be lost sight of entirely. The first thing to be brought before the mind is a mental picture of how that mouth appeared when all those teeth were fully erupted and each tooth in normal condition. This is the "blue print" that must be followed and all work carried on to that end. Individuality can come later. I believe right here is where the greatest possibilities are cut short and only an attempt, if not an entire failure, is experienced. The greatest possibilities cannot otherwise be gained and the importance of this initial step cannot be regarded too seriously.

Ther are many details pertaining to the casting of gold inlays that the writer would have been pleased to discuss, but time will not permit referring to these in a manner they deserve at this time. In brief, however, care in every detail of the technique of casting is the important factor. In fact, the same has been proven to be so. There is no place anywhere throughout the whole procedure of the work that careless methods can be tolerated and the greatest possibilities gained. The two are entire strangers if not sworn enemies. A word should be spoken regarding the strongest objection raised against the inlay principle, and this is in reference to the unreliability of cement. It is true cement is an unstable compound when exposed to the fluids of the mouth. Castings can be made so accurately to fit the cavity that the cement is not discernable to the eye. If this is not our standard and cement lines are exposed, more or less danger is insured, and failure is not due the inlay principle, but must be credited to faulty technique. Perfectly fitting inlays have been too frequently demonstrated to leave any question regarding this point.

In closing, one thought should prevail—accomplish greater possibilities. In the doing of this each of us is the greater for it, those we serve the happier, and our profession the better that we have lived.—*Dominion Journal.*

